# Foreign Body Aspiration Presenting as Pneumothorax in a Child

**DOI:** 10.7759/cureus.8161

**Published:** 2020-05-16

**Authors:** Maxwell D Newby, David Thomas, Charles J Mullett, Chickajajur Vijay, Michele M Carr

**Affiliations:** 1 Otolaryngology, West Virginia University, Morgantown, USA; 2 Pediatrics, West Virginia University, Morgantown, USA; 3 Otolaryngology, Jacobs School of Medicine and Biomedical Sciences, University at Buffalo, Buffalo, USA

**Keywords:** developmental pediatrics, airway disorders, food allergy quality of life, ent procedures, safe retrieval

## Abstract

A typical presentation of a foreign body aspiration (FBA) in a child includes witnessed choking, respiratory distress, cyanosis, coughing, wheezing, diminished breath sounds, and/or altered mental status. Following an extensive literature review, we found pneumothorax occurring secondary to FBA is a rare occurrence and should elicit prompt treatment. This 17-month-old female was admitted for respiratory syncytial virus (RSV) bronchiolitis and developed a subsequent pneumothorax during her hospital stay, consequent to aspiration of a cashew fragment two weeks before presentation. In light of the National Institute of Allergy and Infectious Diseases (NIAID)-sponsored expert panel’s addended guidelines, published and endorsed by the American Academy of Pediatrics (AAP) in 2017, we highlight a potential complication of increasing encouragement of peanut consumption in children as young as four months.

## Introduction

Diagnosing foreign body aspiration (FBA) in children requires a high index of suspicion. Without a witnessed choking episode, patients will often present after failing therapy for otitis media, pneumonia, or asthma. Although FBA is common in the pediatric population, a pneumothorax is a unique consequence [1]. Complications such as atelectasis, pneumonia, and bilateral emphysema have been well-documented in the literature [2,3]. Pneumothorax was first documented by Blazer et al. in 1980 as an extremely rare complication, occurring in less than 1% of patients with a FBA, and subsequently by Berdon et al. in 1984 [4]. Banerjee et al. documented a case of a child aspirating an earring that eroded through the bronchial wall and resulted in a pyopneumothorax, a potential risk in FBA depending on the type of foreign body and the length of time it remains in the airway [5].

Physiologically, a pneumothorax can develop either by the erosion of a foreign object through the bronchial wall into the pleural space or due to acute obstructive emphysema producing a ball-valve effect [6]. Organic material is more likely to be aspirated in children; the most likely culprit in the United States is peanuts, accounting for nearly half of all inhaled foreign bodies. Recently, the American Academy of Pediatrics (AAP) published guidelines advising peanut introduction as early as four months of age for those infants at high risk for peanut allergy [7]. Lack of proper parental education on this topic may lead to an increase in frequency of FBA, specifically peanuts and other nuts.

## Case presentation

A 17-month-old female presented to an outside facility’s emergency department on day 0 with rhinorrhea, unabating cough, repetitive emesis, and two-day history of subjective fevers which the mother had opted not to treat. A week beforehand, the patient visited her primary care provider for rhinorrhea, and the patient’s mother was told this is likely secondary to teething. On the previous day, she had been taken to an urgent care clinic after the development of a fever and was subsequently started on outpatient amoxicillin for presumptive otitis media. The patient had an uneventful past medical history and was born via spontaneous vaginal delivery. A rectal temperature showed a fever of 105.1 °F. Heart rate was 188 bpm and respiratory rate was 54. Physical exam revealed usage of accessory respiratory musculature with retractions, diminished left-sided breath sounds with crackles, and tachycardia. The patient was positive for respiratory syncytial virus (RSV) on a nasopharyngeal swab and negative for a rapid strep test and influenza. A frontal and lateral chest X-ray indicated a slightly hyperinflated left lung with a basilar infiltrate involving the lingula and left lower lobe - lungs were otherwise normal without effusion nor pneumothorax.

**Figure 1:**
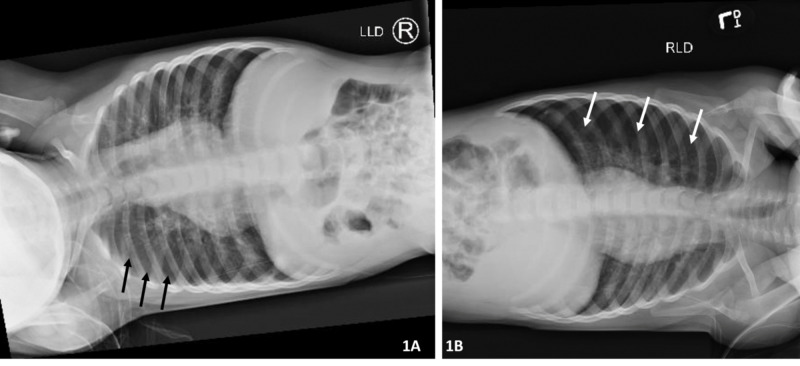
A) Left lateral decubitus X-ray on day four demonstrated air trapping in the left lung without physiological lung collapse. Black arrows outline the lateral aspect of the lung. B) Right lateral decubitus physiological lung collapse with left side pneumothorax. White arrows outline the lateral aspect of the lung

The outside facility’s emergency department recommended beginning ceftriaxone in the setting of RSV bronchiolitis and possible pneumonia; the mother opted to elope with the child to seek a second opinion at a nearby hospital. The patient appeared at the nearby hospital the next morning and was admitted. Lab work indicated a white blood cell count of 19.8 x 103/μL and fever had decreased to 103.5 °F on acetaminophen. The patient received four doses of ceftriaxone over the next four days but demonstrated the development of a worsening pneumothorax on repeat chest X-rays done on day three and day four. The radiologist at the outside hospital recommended left and right lateral decubitus chest X-rays for a possible foreign body and completed it on day three; however, the referring physician inquired about potential hospital transfer for cardiothoracic surgery and potential chest tube placement (Figures 1A-1B). Therefore, the decision was ultimately made to transfer the patient to Ruby Memorial Hospital’s Pediatric Intensive Care Unit on day four.

**Figure 2:**
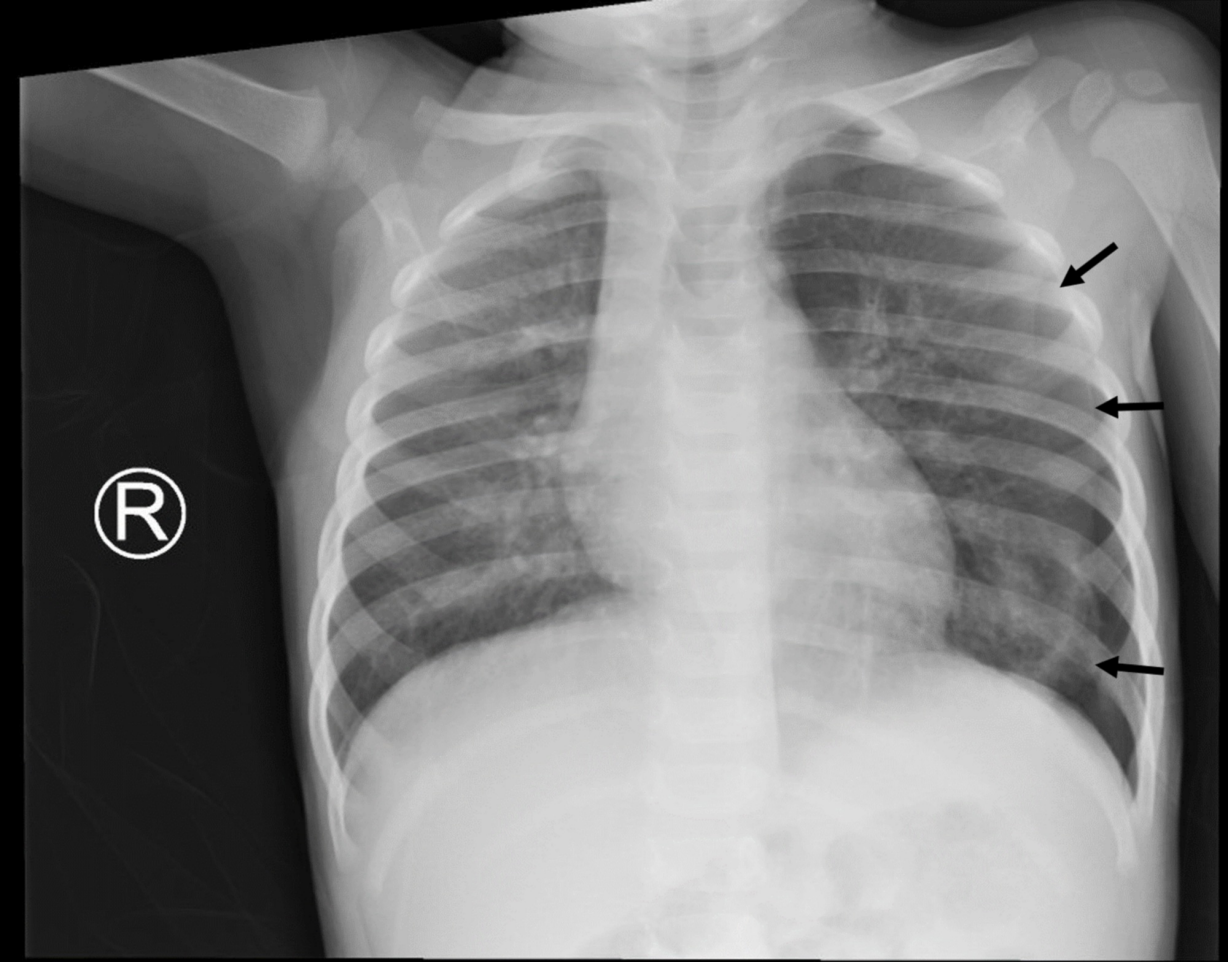
Chest X-ray on day four demonstrated left-sided pneumothorax on arrival from outside facility; black arrows outline the lateral aspect of the lung

On arrival, an anterior posterior chest X-ray exhibited continued left-sided pneumothorax (Figure 2). Patient’s mother declined thoracostomy decompression. Pediatric otolaryngology and pediatric pulmonology were consulted regarding possible foreign body causing a ball-valve defect. Upon further interviewing, the mother recalled an episode about two weeks earlier where patient coughed after being fed cashews by her grandfather. Otolaryngology recommended flexible bronchoscopy to assess underlying pulmonary anatomy; pulmonology recommended deferring flexible bronchoscopy to monitor recovery from respiratory infection.

**Figure 3:**
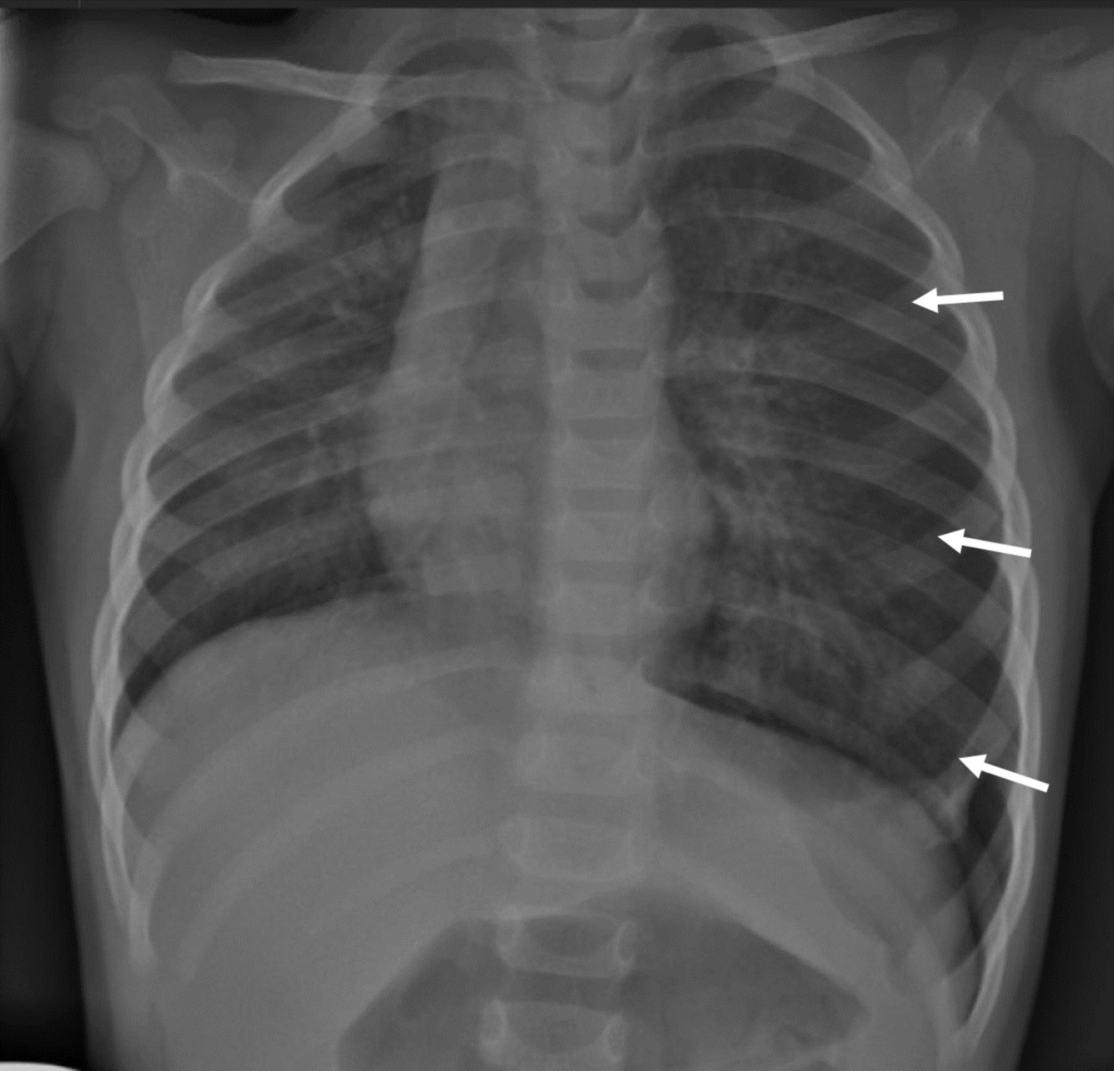
Chest X-ray on day five demonstrated increased left-sided pneumothorax with a mediastinal shift, despite the patient’s clinical improvement; white arrows outline the lateral aspect of the lung

**Figure 4:**
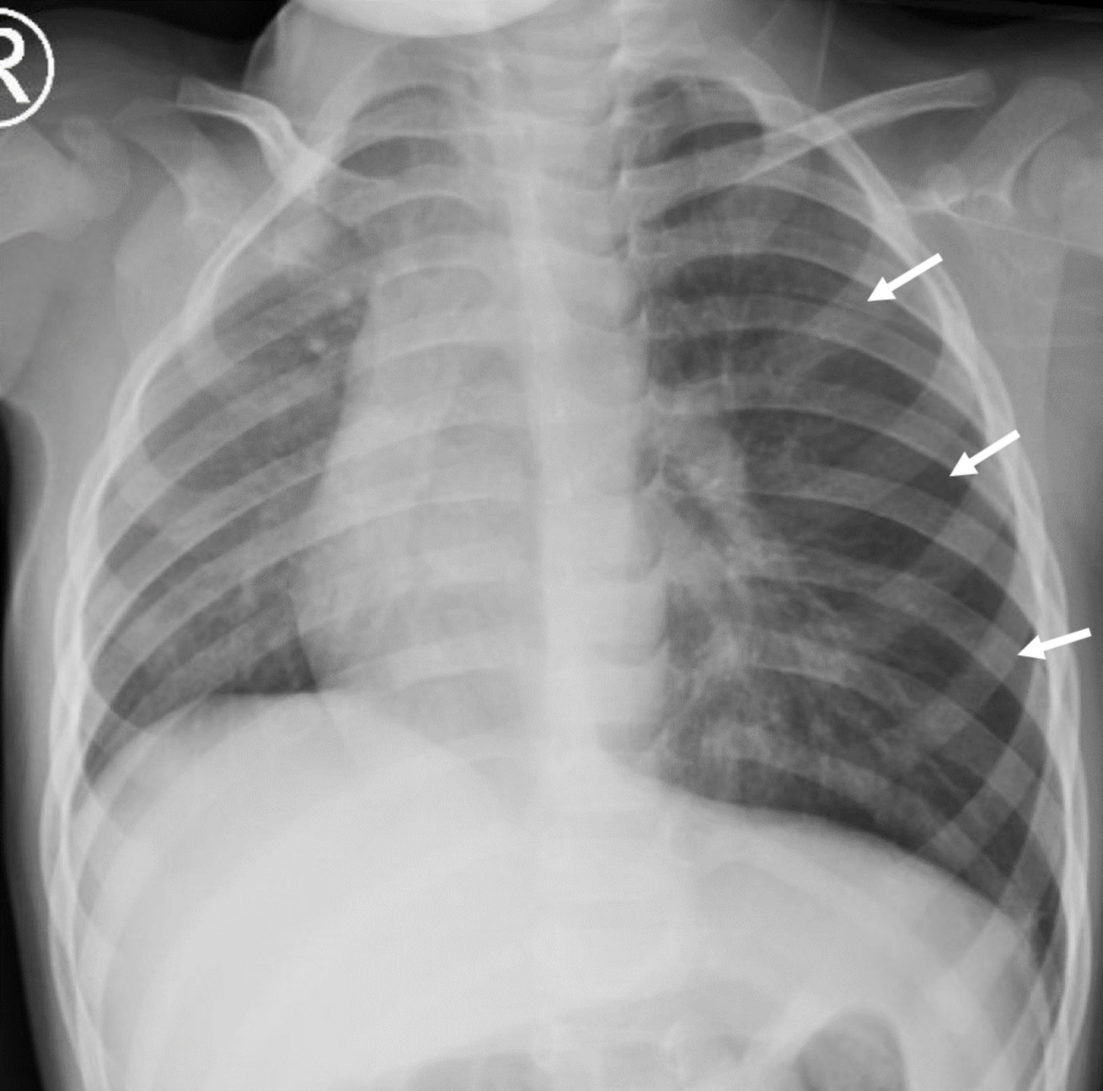
Chest X-ray on day six demonstrated stable left-sided pneumothorax; white arrows outline the lateral aspect of the lung

Patient was treated with intravenous acetaminophen and 100% oxygen via non-rebreather mask. By day five, patient’s respiratory status improved, patient was afebrile, and white cell count returned to normal limits. Imaging showed increased size of pneumothorax with a deep sulcus sign and a right midline shift (Figure 3). Patient was weaned to room air and transferred to general pediatric service. Imaging on day six showed continued left pneumothorax with possible atelectasis (Figure 4). Physical exam continued to show decreased left-sided breath sounds. Shared decision between the patient’s mother, primary pediatric team, and the consulting services culminated in a direct laryngoscopy, rigid bronchoscopy, and flexible bronchoscopy on day eight.

**Figure 5:**
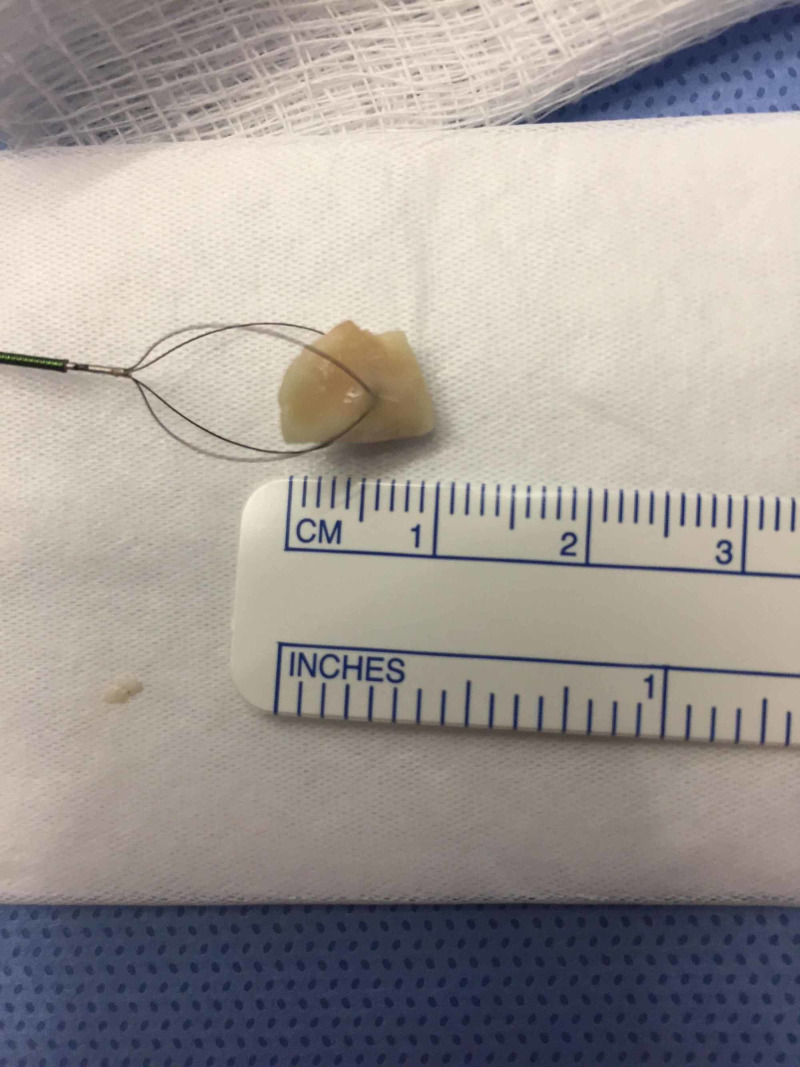
Foreign body removed from left mainstem bronchus en bloc - a 0.8 x 0.7 x 0.4 cm cashew nut

Direct laryngoscopy revealed grossly normal oropharyngeal anatomy to the level of the glottis. Subsequent rigid bronchoscopy displayed significant secretions at the carina emanating from the left mainstem bronchus. Rigid bronchoscopy identified a foreign object, assumed to be a nut, in the left mainstem bronchus. A 12-mm basket was used to remove the foreign object en bloc - a 0.8 x 0.7 x 0.4 cm cashew nut (Figure 5). Rigid bronchoscopy was used to survey both mainstem bronchi and the trachea. A fragment of the foreign object was identified on the tracheal wall and retrieved with optical forceps. The pediatric pulmonology team then performed a flexible bronchoscopy to survey the smaller bronchioles for other foreign objects and none were found.

**Figure 6:**
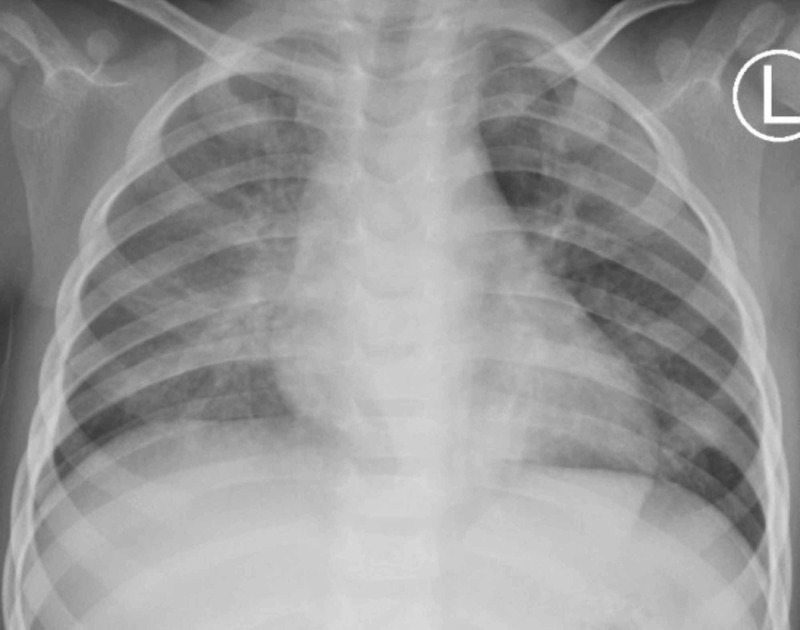
Chest X-ray on first post-operative day demonstrated resolution of pneumothorax

The patient recovered quickly. Clinically, the patient’s mother reported episodes of coughing decreased significantly. On physical exam, lung sounds were symmetric. Imaging on the morning of day nine demonstrated resolution of the pneumothorax and right midline shift (Figure 6). The patient was discharged on day nine and scheduled for follow-up with the pediatric pulmonary team.

## Discussion

FBA is a common cause of serious illness, and possible death, in children. According to the National Safety Council, mechanical suffocation was the fourth leading cause of preventable fatality in the United States in 2018, accounting for 6,701 deaths across all age groups. In children between the ages of one and four, mechanical suffocation rises to the third leading cause of preventable fatality at 123 deaths, behind only motor vehicle accidents and drownings [8]. Prompt diagnosis and retrieval of the foreign body is fundamental to prevent respiratory complications and possible death.

Complications of FBA are numerous including atelectasis, pneumonia, bilateral emphysema, and pneumothorax [9]. This case report provides a challenging aspect of FBA as the diagnosis was made more difficult due to the lack of a witnessed choking episode and the existence of multiple alternate diagnoses. The delayed presentation of a left pneumothorax, in this case secondary to a nut lodged in the left mainstem bronchus believed to be aspirated more than two weeks prior, cautions health care providers to elicit historical data beyond the immediate time period.

In the setting of a radiolucent aspirated airway foreign body, radiographs may be helpful in demonstrating only indirect signs such as air trapping or atelectasis. These radiographic findings may also be falsely negative in 10%-30% of cases [9]. In particular, the use of lateral decubitus X-rays may be helpful in younger children who are unable to perform or cooperate with inspiratory and expiratory films. Pertinent positive findings that would indicate an airway obstruction and air trapping would be the absence of lung collapse in the affected lung on the ipsilateral side. Lung collapse occurs due to on-going alveolar disruption caused by ball-valve obstruction by the foreign body. Unfortunately, these classic findings are historically unreliable and often falsely negative as the cooperation of the child and the skill of the technician are often variable. In this case report, the lateral decubitus X-rays, as seen in Figures 1A-1B, were positive for concern for an airway foreign body and essential to the diagnosis of this child’s case; therefore, this type of study should remain a crucial portion of work-up for concern of an airway foreign body.

In 2017, the AAP endorsed the National Institute of Allergy and Infectious Diseases’ (NIAID) guidelines advising peanut introduction as early as four months of age for those infants at high risk for peanut allergy. These recommendations, stemming from the Learning Early About Peanut Allergy (LEAP) trial, overturned previous 2000 guideline recommendations of no peanut consumption until the age of three [7]. Although the AAP specifically recommends “infant-safe forms of peanuts”, such as creamy peanut butter, to reduce the risk of peanut allergies, caregivers are commonly unaware what this means for their child. Molar teeth, which erupt around 14 to 18 months of age, are fundamental to allow for proper mastication of foods such as peanuts; therefore, children between the age of one and two are in a critical period where caretakers can mistakenly assume the child is capable of eating hard food, including nuts, that requires effective chewing. Furthermore, children between the ages of one and two have a tendency to place objects in their mouth for the sake of discovery. Therefore, parents should be counseled that solid peanuts should not be introduced into children’s diets until their molar teeth have properly erupted.

## Conclusions

FBA warrants prompt medical treatment. Prolonged retention of a foreign body may lead to bronchial reaction with subsequent pneumonia, atelectasis, or pneumothorax. In young children, FBA typically involves organic material - specifically peanuts. Given the AAP’s 2017 guidelines encouraging parents to introduce peanuts to their children’s diet early, it is incumbent that physicians properly educate parents on safe methods of doing so. For children, this means no solid peanuts until full eruption of molar teeth.
